# Daidzein is the *in vivo* active compound of Puerariae Lobatae Radix water extract for muscarinic receptor-3 inhibition against overactive bladder

**DOI:** 10.3389/fphar.2022.924251

**Published:** 2022-10-04

**Authors:** Yining Qiang, Lu Bai, Shuran Tian, Yi Ma, Pingxiang Xu, Mingchang Cheng, Yi Wu, Xiaorong Li, Ming Xue, Xuelin Zhou

**Affiliations:** ^1^ Department of Pharmacology, School of Basic Medical Sciences, Capital Medical University, Beijing, China; ^2^ Beijing Engineering Research Center for Nerve System Drugs, Beijing, China

**Keywords:** overactive bladder, Puerariae Lobatae Radix, *in vivo* active components, bioassay-guided fractionation, muscarinic receptor inhibition

## Abstract

**Background:** In the previous study, Puerariae Lobatae Radix (named Gegen in Chinese) water extract attenuated M3 receptor agonist carbachol-induced detrusor contraction after 3-week oral administration in a hypertension-associated OAB (overactive bladder) model. This research aimed to investigate the active ingredients from Gegen water extract against OAB.

**Methods:** Bioassay-guided fractionation was performed by using preparative HPLC for fast isolation of fractions followed by screening their *ex vivo* activity through carbachol-induced bladder strip contraction assay. Chemicals in each active fraction were analyzed by HPLC-UV. Urine metabolites were quantified by LC-MS/MS after sub-acute administration. Thermal shift assay with the recombinant human M3 receptor protein was performed, and molecular docking analysis was used for molecular modelling of M3 receptor inhibition.

**Results:** Bioassay-guided fractionation results for isolating M3 receptor inhibitors indicated that four compounds were identified as active ingredients of Gegen water extract, and their inhibition potency on carbachol-induced detrusor contraction was ranked in descending order according to their inhibition concentrations as follows: genistein > daidzein > biochanin A >> puerarin. Daidzein in urine reached an *ex vivo* effective concentration to inhibit detrusor contraction, but others did not. Daidzein concentration-dependently increased the melt temperature (*T*m) of recombinant human M3 receptor protein with a positive binding (Δ*T*m = 2.12 °C at 100 μg/ml). Molecular docking analysis showed that daidzein can potently bind to the ligand binding pocket of the M3 receptor via hydrogen bonding.

**Conclusion:** Puerarin and its derivatives were pro-drugs, and daidzein was their *in vivo* active form via M3 receptor inhibition for treating OAB.

## Highlights

• The major components of Gegen water extract were isoflavone glycosides (puerarin and its derivatives), but their aglycones were the potent M3 receptor inhibitors as found by bioassay-guided fractionation.
• The contents of aglycones (e.g., daidzein) as the major urine metabolites were quantified by LC-MS/MS.
• Daidzein can bind to recombinant human M3 receptor as revealed by thermal shift assay, but peurarin did not.
• Puerarin and its derivatives were pro-drugs.

## 1 Introduction

Overactive bladder (OAB), a common disease, is defined as a kind of clinical syndrome followed by urinary dysfunction and urgency, with or without incontinence, often accompanied by increased urinary frequency and nocturia (White and Iglesia, 2016). It could seriously affect the patients’ quality of life and disturb their normal sociality, and it is so-called “social cancer.” The global prevalence of OAB was about 9–43% in women and 7–27% in men (Milsom et al., 2014; Przydacz et al., 2020). Clinical investigation showed that OAB gradually increased with ageing, and the overall prevalence rate of people over 40 years old in China was 11.3% (Chen et al., 2015; Zhu et al., 2015). The pathogenesis of OAB is generally believed to be the result of many factors. At present, one of the main pathogenesis theories of OAB is myogenic, which includes an abnormal excitation of bladder smooth muscle (Andersson et al., 2015).

Selective muscarinic receptor-3 (M3) inhibitors (e.g., solifenacin and darifenacin) are the first-line drugs for treating OAB in clinics (Zinner et al., 2006). However, side effects such as dry mouth and constipation become serious after their long-term use (Yang et al., 2021). The M3 receptor is located in the bladder mainly for controlling detrusor contraction, which is activated by urothelium-released acetylcholine. Thus, drugs in urine possibly act on urothelium for OAB treatment (Ito et al., 2016). Bladder infusion refers to injecting drugs directly into the bladder through a catheter to maximize their local effects. Intravesical administration of oxybutynin hydrochloride, an anti-muscarinic drug, is a potential second-line treatment for patients that cannot tolerate oral anticholinergic drugs (Shen et al., 2022). However, its intravesical delivery still needs a frequent and cumbersome process in clinics. Therefore, it is urgent to develop an alternative treatment with low side effects for treating OAB.

**FIGURE 1:**
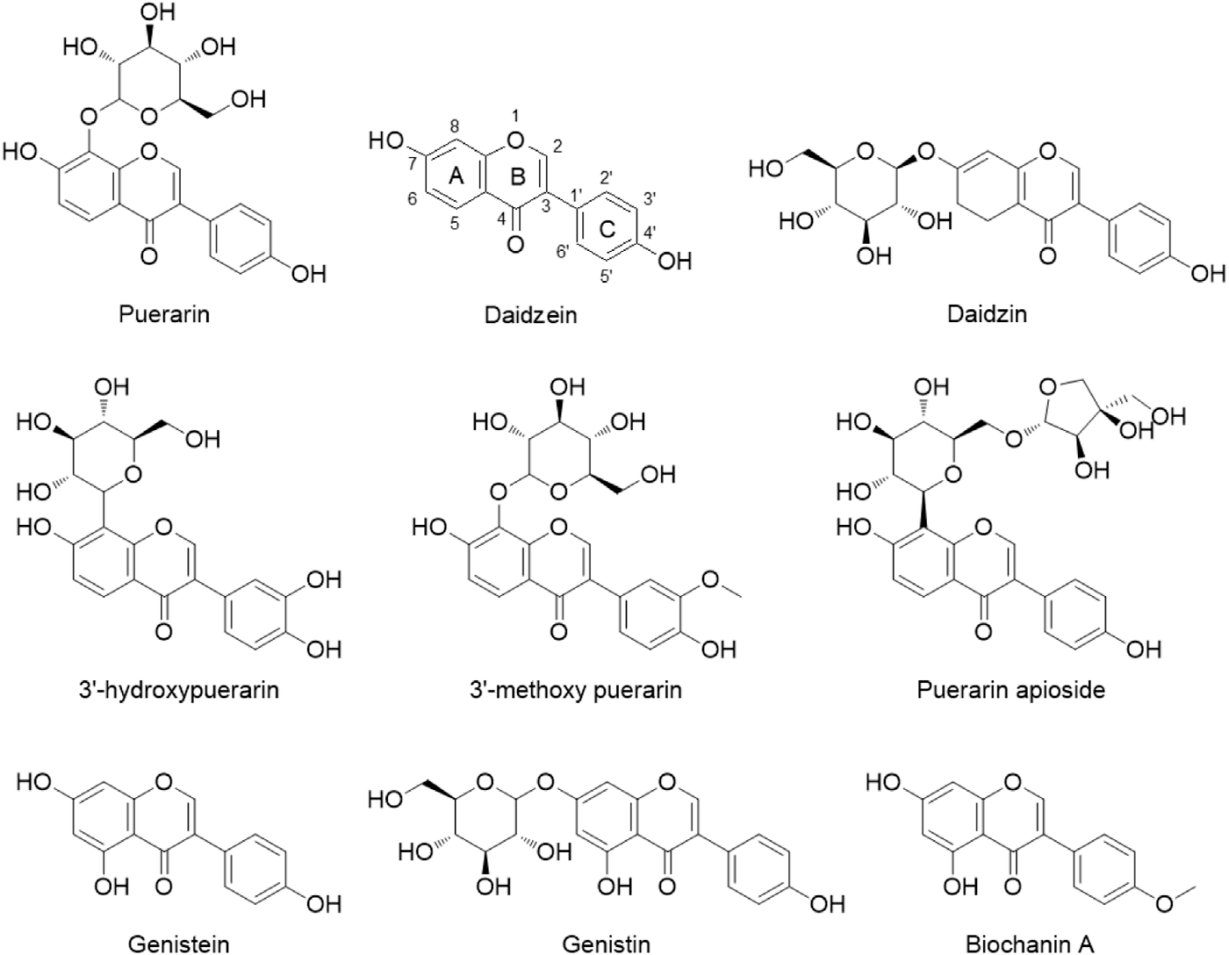
Structures of the major components in Gegen water extract.

As a common functional food and well-tolerated medicinal herb, Puerariae Lobatae Radix (named Gegen in Chinese) has been used for medicinal purposes and daily soup for hundreds of years in China and other regions of East Asia. Gegen is honored as “longevity powder” or “Asian ginseng” due to its high nutritional values and high safety (Zhang et al., 2020). The major components in Gegen water extract are isoflavone glycosides such as puerarin (daidzein-8-C-glucoside) and its glycosides, daidzin (daidzein-7-O-glucoside), and genistin (genistein-7-O-glucoside), and the contents of liposoluble aglycones like daidzein were low due to water extraction (Figure 1) (Zhang H. et al., 2019). Gegen and its major component puerarin have been reported for their relaxation effect through different mechanisms on the basilar artery (Prasain et al., 2004), pulmonary arterial smooth muscle (Zhang X. et al., 2019), and microvessels (Deng et al., 2012). In the previous study, the *ex vivo* analysis of detrusor functions showed that after 3-week oral administration, Gegen water extract at 300 mg/kg, a clinical equivalent dose, decreased detrusor tonic contraction and phasic frequency stimulated by muscarinic receptor agonist carbachol; meanwhile, it was also able to reduce tonic contraction caused by electric field stimulation (Zhou et al., 2016). These results indicated that Gegen water extract could improve detrusor overactivity through anti-muscarinic mechanisms. However, it was still unknown which compounds were responsible for this inhibition on muscarinic receptor.

Pharmacokinetics and metabolism of major compounds in Gegen water extract have been reported. After oral administration of Gegen water extract containing low content of aglycones (Puerarin: daidzin: daidzein = 6.42:1:0.14, w/w/w ratio), it was reported that plasma AUCs of puerarin and daidzein were 15.1 μg·h/mL and 4·4 μg·h/mL, respectively, showing that daidzein was its major metabolites in plasma (Wei et al., 2020). The contents of chemicals in the urine after sub-acute treatment of Gegen water extract were still unknown, although there was a report for its urine metabolite profile. Similar to intravesical oxybutynin, pro-drugs and metabolites of Gegen water extract in the urine probably acted on M3 receptors in the bladder wall if their concentrations can meet the threshold of their inhibitory effects. Hence, our study aimed to identify the pharmacological compounds from Gegen water extract that were responsible for the inhibition of muscarinic receptor-3 against OAB.

## 2 Methods and materials

### 2.1 Chemicals and materials

Commercially obtained standards (purity >98%; HPLC grade) of puerarin, 3′-hydroxypuerarin, 3′-methoxy puerarin, puerarin apioside, genistin, daidzein, and genistein were purchased from Chengdu Chroma Co. (Chengdu, China). Carbachol, SYPRO Orange protein stain (5000x), and other unspecified chemicals were purchased from Sigma-Aldrich Co. LLC (St. Louis, MO, United States). Darifenacin hydrobromide was provided by Amandas (Shanghai, China). Full-length recombinant human M3 muscarinic acetylcholine receptor membrane preparation was provided by Millipore Co. (Merck KGaA, Darmstadt, Germany). The composition used for preparing Krebs’ solution was as follows: NaCl, 119 mM; KCl, 4.6 mM; MgCl_2_, 1.2 mM; NaH_2_PO_4_, 1.2 mM; NaHCO_3_, 15 mM; CaCl_2_, 1.5 mM; and D-glucose, 11 mM.

### 2.2 Herbal extraction

The dried root of Pueraria lobata (Willd.) Ohwi (harvested in Henan Province; Lot No. 17072102) was purchased from Beijing Lvye Pharmaceutical Co (Beijing, China). According to Chinese Pharmacopoeia v.2015, the supplier chemically authenticated the raw herb with reference herb and chemical marker puerarin using thin-layer chromatography, showing its good quality as required. The raw materials (400 g) were extracted by boiling with water (4 L) for an hour and repeated once. After filtering, the supernatant was collected and subjected to freeze-drying for the dried extract, which was stored in the desiccator before use.

### 2.3 Chemical profile of Gegen water extract

The chemical composition of Gegen water extract was analyzed via the Agilent 1100 Infinity HPLC-UV system (Santa Clara, CA, United States) after the dried powder was dissolved in water and filtered using a 0.45-μm filter according to our previous study (Zhou et al., 2016). The content of each compound in Gegen water extract was listed as follows (mean ± SEM): puerarin, 221.3 ± 8.34 μg/mg; daidzein, 8.07 ± 0.01 μg/mg; 3′-methoxypuerarin, 39.59 ± 0.81 μg/mg; 3′-hydroxypuerarin, 72.98 ± 3.10 μg/mg; puerarin apioside, 64.29 ± 2.78 μg/mg; and daidzin, 41.03 ± 1.32 μg/mg (Supplementary Figures S1A and S1B).

### 2.4 Animals

The nine-week-old male spontaneously hypertensive rats (SHRs) were provided by Beijing Charles River Laboratory Animal Technology Co., Ltd. To lower the estrogen effect of Gegen isoflavones, male rats were used in this study. All experiments were approved by the Animal Ethics Committee of Capital Medical University (Approved Ethics Number AEEI-2018-098). Animals got free access to soybean-free chow (Beijing Ke-Ao-Xie-Li Co., China) and water for 3 weeks during drug treatment.

### 2.5 Carbachol-induced bladder strip contraction for *ex vivo* activity screening

SHRs were sacrificed with carbon dioxide, and the whole bladder was immediately isolated to prepare detrusor strips in Krebs’ solution. Then, the strips were placed in a PanLab organ bath (Harvard Apparatus, Holliston, Massachusetts, United States) full of bubbling Krebs’ solution at 37°C. After stimulation with high-potassium solution, the strips were washed with Krebs’ solution three times. When the strips were incubated in Krebs’ solution until the tension was set at about 1.5 g, 0.1% DMSO was added for a 10-min incubation as a control, and then the muscle strip was stimulated to shrink by adding muscarinic agonist carbachol (10 μM), which is used for determinization of M3 receptor function in the bladder strip (Liang and Leung, 2012). Between two stimulations, the strips were washed out and balanced for 15 min. The contraction force of the bladder strip was recorded. After the experiment, detrusor strips were collected, dried, and weighed for data normalization. Data were recorded and analyzed using LabChart software (version 8.0).

### 2.6 Bioassay-guided fractionation

Bioassay-guided fractionation was performed by using preparative HPLC for fast isolation of fractions and carbachol-induced bladder strip contraction for *ex vivo* activity screening. Briefly, 10 g of Gegen water extract was totally dissolved in 10 ml of double distilled water and then mixed with 190 ml of ethanol. The mixture was put on the magnetic agitator for stirring and performed ultrasonication for 30 min to precipitate the fiber and protein, etc. After being filtered through a 0.45-μm filter membrane, the purified filtrate was transferred into a 250-ml flask. The liquid was drained using a rotary evaporator at 60°C and a rotating speed of 110 rpm under vacuum, and the dried samples were collected. The dried extract was totally dissolved in methanol (5 ml) and filtered through a 0.45-μm filter membrane into the sample vial for injection to the Agilent 1260 preparative HPLC. An Agilent Zorbax Eclipse XDB-C18 preparative column (21.2 × 150 mm, 5 μm) was used, and the flow rate was set at 20 ml/min. The fractionation was performed through two different mobile phases. The mobile phase for the first fractionation consisted of solution A (distilled water) and solution B (acetonitrile) with the following gradient: 10–12.5% B from 0 to 10 min; 12.5–15% B from 10 to 15 min; 15–60% B from 15 to 20 min; 60–90% B from 20 to 25 min; and 90% B from 25 min to 30 min. The mobile phase for the second fractionation consisted of solution A (distilled water) and solution B (acetonitrile) with the following gradient: 10–30% B from 0 to 10 min; 30% B from 10 to 15 min; 30–60% B from 15 to 20 min; and 90% B from 20 min to 30 min.

The relevant components were separated and enriched according to their chemical profiles in HPLC chromatograms using the Agilent 1100 HPLC system. The HPLC mobile phase consisted of solution A (0.1% acetic acid) and solution B (acetonitrile) with the following gradient: 10–12.5% B from 0 to 20 min; 12.5–15% B from 20 to 30 min; 15–60% B from 30 to 40 min; and 60–90% B from 40 min to 45 min. The HPLC elution was performed using an Alltima HPLC C18 column (250 mm × 4.6 mm, 5 μm) guarded using a guard column with the same stationary phase. The column was maintained at room temperature, and the flow rate was set at 1 ml/min. The UV absorbance was detected at 254 nm, and the injection volume was 10 μl. The fractions with similar HPLC-UV profiles were combined and transferred into the 250-ml flask for rotary evaporation to collect dried fractions using the same temperature and speed as mentioned previously.

Detrusor strips were isolated from SHRs and placed in the organ bath as mentioned previously, and then inhibitory activities of different fractions at 100 μg/ml were tested under carbachol (10 μM)-stimulated detrusor contraction. The chemical profiles of potent fractions were analyzed by HPLC-UV at 254 nm with authentic compounds. After confirming potent compounds in each fraction, the concentration-dependent inhibitory effect of each potent compound (6.25-50 or 100 μg/ml in 0.1% DMSO-Krebs’ solution) against carbachol (10 μM)-induced detrusor contraction was performed. Darifenacin (12.5–200 ng/ml) served as a positive control.

### 2.7 Determination of urine metabolites

When the last dose of Gegen water extract (300 mg/kg) in a 3-week treatment was given to SHRs, conscious rats were put into the metabolic cages with free access to water. Urine samples were collected into ice-cold 15-ml Falcon tubes every 2 h and up to a total of 8 h, and the tubes were cooled by ice to prevent bacteria proliferation and water evaporation during collection. The urine of each rat was first centrifuged at 13,000 rpm at 4°C for 10 min, and the supernatant was collected. Then, 50 μl of urine and 150 μl of methanol were added into the centrifuge tube for fully precipitating protein and then centrifuged at 13,000 rpm and 4°C for 10 min to collect the supernatant for sample analysis via UPLC-MS/MS.

Urine metabolites were analyzed by ultra-performance liquid chromatography (UPLC, Waters) coupled with a Xevo-TQS mass spectrometer (Waters) using the Acquity UPLC HSS T3 column (2.1 mm × 150 mm, 1.8 μm). The mobile phase flow rate was 0.4 ml/min and consisted of 0.1% formic acid in water (A) and acetonitrile (B). The gradient profile was set as follows: 10–40% B from 0 to 11 min; and 40–80% B from 11 to 15 min. The parameters for mass spectrometry were as follows: capillary, 2.5 kV for negative mode; sampling cone, 34 V; source offset, 80 V; cone gas flow, 150 L/h; desolvation gas flow, 650 L/h; and desolvation temperature, 350°C. Samples were kept at 4°C in the sample manager, and the column was maintained at 40°C. Extracted ion chromatograms were used to quantify puerarin (m/z 414.94→266.9), daidzein (m/z 252.96→90.91), genistein (m/z 268.96→132.93), and biochanin A (m/z 282.98→267.91) in the ESI negative-ion mode. According to the peak area and respective concentrations, the linear ranges of puerarin, daidzein, genistein, and biochanin A were 0.78–50 μg/ml, 0.78-50 μg/ml, 0.16–10 μg/ml, and 6.25–400 ng/ml, respectively (*R* ^2^ > 0.99). The precision and accuracy of the quantitation were satisfied according to FDA Bioanalytical Method Validation Guidance for Industry. Total ion chromatograms including retention times and MRM masses are shown in Supplementary Figure S2.

### 2.8 Thermal shift assay for the potential binding to M3 receptor

A thermal shift assay was performed to measure the binding of ligands to a recombinant human M3 receptor as modified from the previous study (Huynh and Partch, 2015). Briefly, after optimization of protein and dye concentrations, the M3 receptor protein (0.025 mg/ml) was incubated with different concentrations of tested compounds (0–100 μg/ml in 1% DMSO) in an 18 μl volume of ice-cold PBS for 2 h. Then, SYPRO Orange protein stain (2 μl; 8x as the final concentration diluted from original 5000x with PBS) was added, and the melt curve was detected using the Applied Biosystems QuantStudio5 real-time PCR system under the following conditions: ramp speed, standard; excitation wavelength at 470 nm (Reporter: FAM), and emission wavelength at 586 nm (Quencher: TAMRA) with no passive reference; an initial 2-min hold at 25°C (ramp rate, 1.6°C/s), ramping up in increments of 0.05°C/s to a final temperature of 99°C. Melt temperatures (*T*m) were calculated using Protein Thermal Shift software v.1.4 (Applied Biosystems, Thermo, United States). The change in melt temperature (Δ*T*m) was equal to the *T*m of control (1% DMSO) subtracted from the *T*m of the tested compound. Δ*T*m higher than 2°C was considered a positive interaction between the ligand and receptor protein (Huynh and Partch, 2015).

### 2.9 Molecular docking analysis for molecular modelling

To better understand the binding affinity of chemicals to the M3 receptor and comprehensively explain the structure–activity relationship of these isoflavones, molecular docking was performed for modelling. AutoDock Vina v.1.0.2 software was used for the molecular docking analysis, and the software parameters were set by default (Trott and Olson, 2010). The protein crystal structure of the M3 receptor was selected from the Protein Data Bank. The molecular docking conformation was further optimized by selecting the binding mode of the compound to the M3 receptor (PDB ID 5ZHP) with the lowest binding free energy (Liu et al., 2018). The crystal structure of the M3 receptor was used to conduct virtual docking of the aforementioned compounds, and the potential active chemical components were determined by comparison with the positive control (crystal ligand 9EC). Simulation results were output and illustrated by LigPlot + v.2.2 (http://www.ebi.ac.uk/thornton-srv/software/LIGPLOT/) (Laskowski and Swindells, 2011) and PyMOL Molecular Graphics System (da Silva Giotto et al., 1998) v.1.3 (Schrödinger, LLC, New York City, United States), respectively.

### 2.10 Data analysis

Data were represented as mean ± standard error of mean (SEM). A non-linear regression analysis was used to calculate the IC_50_ value by using Prism version 5.0 (GraphPad Software, CA, United States). Data were analyzed by one-tailed student *t*-test. When the *p*-value was less than 0.05, the difference was considered statistically significant.

## 3 Results and discussion

### 3.1 Bioassay-guided fractionation

**FIGURE 2:**
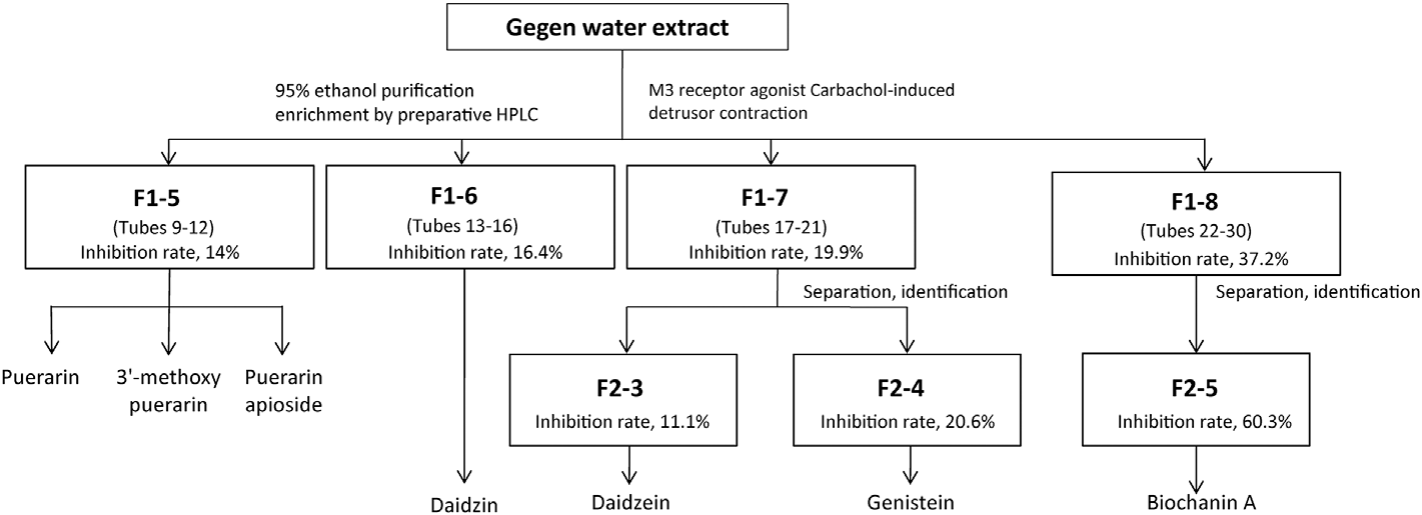
Schematic diagram indicates the fast isolation of active fractions from Gegen water extract using bioassay-guided fractionation with preparative HPLC. Active fractions were selected for further fractionation.

To confirm the potent M3 receptor inhibitors in Gegen water extract, bioassay-guided fractionation was performed by using preparative HPLC and *ex vivo* detrusor contraction assay. The schematic diagram of fractionation is shown in Figure 2. For the first fractionation by the preparative HPLC, 30 fractions were attained from the elution at each minute and combined into 8 fractions according to their similar HPLC-UV chromatograms. Then, these 8 fractions were dried by rotary evaporation as mentioned previously and subjected to *ex vivo* pharmacological tests, respectively (Supplementary Figures S1C and S1D).

Fraction F1-5 (combined tubes 9–12 of first fractionation) with an inhibition rate of 14% contained puerarin, 3′-methoxypuerarin, and puerarin apioside, which were identified by authentic standards. Fraction F1-6 (combined tubes 13–16 of first fractionation) was identified as a daidzin-rich fraction with an inhibition rate of 16.4%. According to the composition of the mobile phase, Fraction F1-7 (combined tubes 17–21 of first fractionation) was separated into F2-3 (tube 4 of second fractionation) and F2-4 (tube 5 of second fractionation), which were identified as a daidzein-rich fraction (inhibition rate: 11.1%) and a genistein-rich fraction (inhibition rate: 20.6%), respectively (Supplementary Figures S1E and S1F).

Fraction F2-5 (combined tubes 6–30 of second fractionation) significantly inhibited carbachol-induced detrusor contraction, which suggested that F2-5 contained active substances with an inhibition rate of 60.3%. According to the retention time of authentic standard in the HPLC-UV chromatograms, biochanin A was one of the compounds in Fraction F2-5 (Supplementary Figure S1G).

### 3.2 Inhibition activity of potent active compounds

**FIGURE 3:**
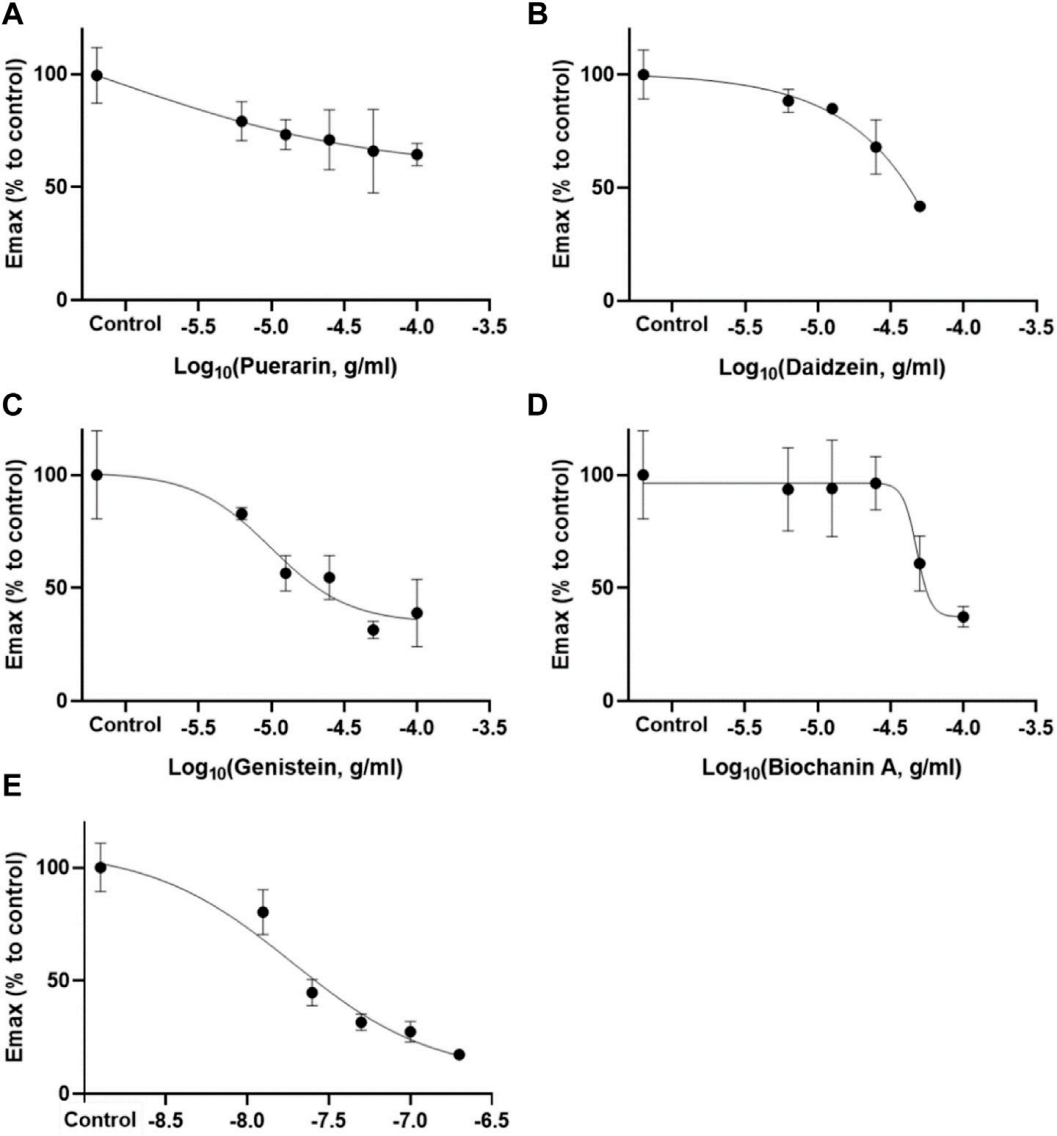
Inhibitory effects of puerarin **(A)**, daidzein **(B)**, genistein **(C)**, and biochanin A **(D)** at different concentrations (6.25–50 or 100 μg/ml) against carbachol (10 μM)-induced detrusor contraction (Mean ± SEM; n = 3). Darifenacin **(D)** (12.5–200 ng/ml) served as the positive control.

According to the retention times of the pure compounds, the major components in each active fraction were identified. Seven compounds were identified, and their inhibition potency on carbachol-induced detrusor contraction was ranked in descending order according to their IC values as follows: genistein (IC_50_ = 18.57 ± 11.20 μg/ml) > daidzein (IC_50_ = 40.27 ± 0.46 μg/ml) > biochanin A (IC_50_ = 57.07 ± 12.47 μg/ml) >> puerarin (IC_10_ = 2.90 ± 2.11 μg/ml) (Figure 3 and Table 1). 3′-Hydroxypuerarin (remaining activity at 100 μg/ml: 82.3 ± 1.8%), 3′-methoxypuerarin (remaining activity at 100 μg/ml: 80.1 ± 3.4%), and puerarin apioside (remaining activity at 100 μg/ml: 89.3 ± 9.2%) showed weak inhibition. Compared with the high inhibition rate of the positive control (darifenacin), biochanin A, daidzein, and genistein showed strong inhibitory effects. It has been reported that genistein was a potent inhibitor against carbachol stimulation in the previous study (Di Salvo et al., 1993), which is consistent with our current results. This is the first time to report the inhibitory activity of biochanin A on M3 receptor.

**TABLE 1:** Inhibition values of active compounds isolated from Gegen water extract on carbachol-induced detrusor contraction (Mean ± SEM, n = 3).

Compound	IC_50_ value (μg/ml)	IC_10_ value (μg/ml)
Daidzein	40.27 ± 0.46	8.15 ± 1.85
Puerarin	ND	2.90 ± 2.11
Genistein	18.57 ± 11.20	5.05 ± 0.58
Biochanin A	57.07 ± 12.47	34.94 ± 6.75
Darifenacin^a^	0.0293 ± 0.0047	0.0076 ± 0.0013

a positive control. ND: not determined.

### 3.3 Quantitation of major metabolites in urine

**FIGURE 4:**
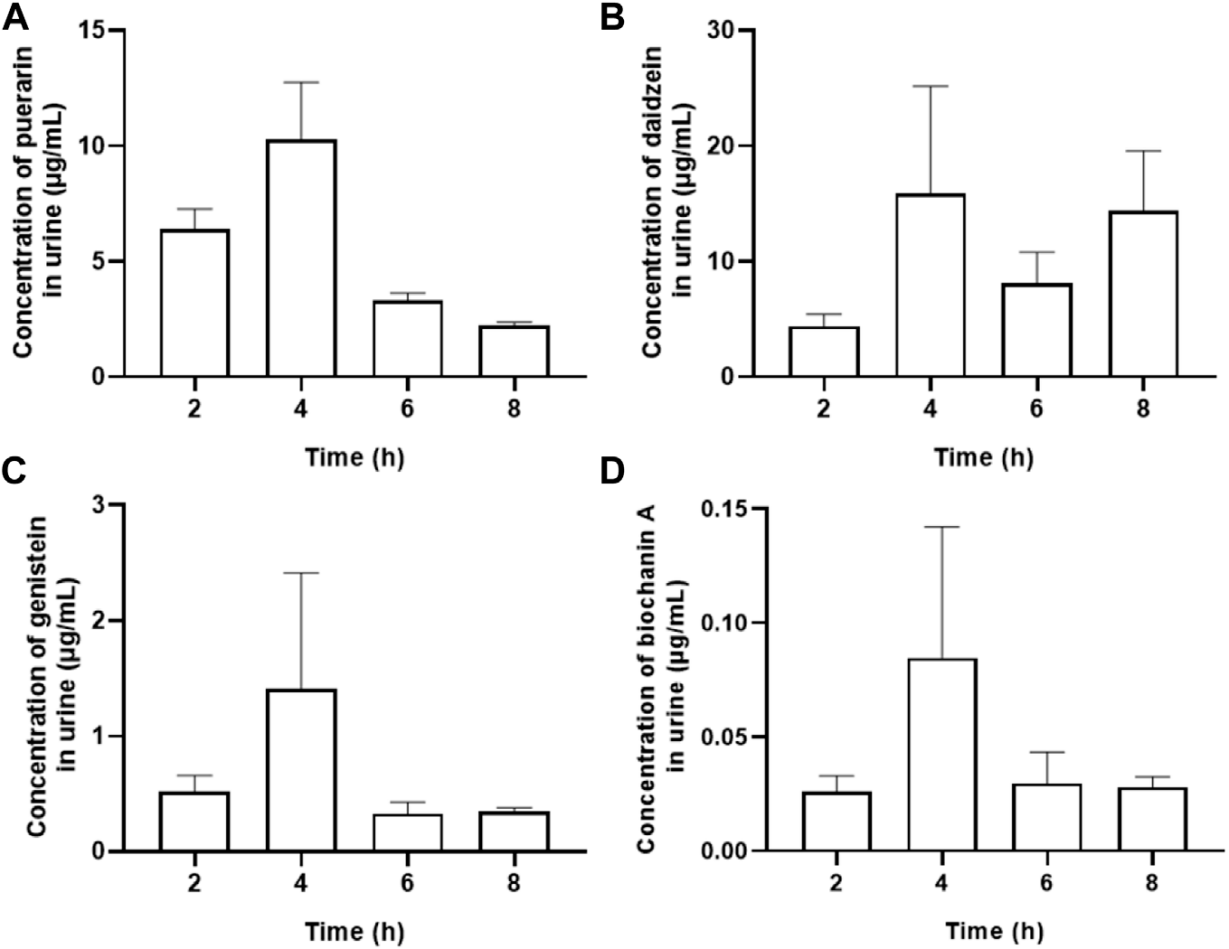
Contents of major urine metabolites including peurarin **(A)**, daidzein **(B)**, genistein **(C)** and biochanin A **(D)** after oral administration of Gegen water extract in SHR as detected by LC-MS/MS (Mean ± SEM; n = 8).

Muscarinic receptor 3 exists in bladder urothelium and sub-urothelial myofibroblasts, suggesting that active chemicals in urine could act on the M3 receptor in urothelial sensory function. The main urine metabolites after oral administration of Gegen water extract were daidzein and other isoflavone glycosides, and the highest concentration of each compound at 4 h was determined as follows (Mean ± SEM): puerarin, 10.280 ± 2.475 μg/ml; daidzein, 15.877 ± 9.303 μg/ml; genistein, 1.410 ± 1.003 μg/ml; and biochanin A, 0.085 ± 0.057 μg/ml (Table 2 and Figure 4). Our results were consistent with the previous study, which showed that daidzein was one of the major urine components of Gegen water extract in a clinical trial (Prasain et al., 2004; Jung et al., 2014; Shang et al., 2017). The content of daidzein in urine reached its IC_10_ value; thus, daidzein in urine could have a direct effect on reducing detrusor contraction with 15% inhibition at 12.5 μg/ml on M3 receptor *ex vivo* (*p* < 0.05). In addition, Gegen water extract did not alter the expression of the M3 receptor as detected by immunochemistry (Supplementary Figure S3).

**TABLE 2:** Contents of major urine metabolites after oral administration of Gegen water extract in SHR detected by LC-MS/MS (Mean ± SEM; n = 8).

Compounds	Content (μg/ml)			
	2 h	4 h	6 h	8 h
Puerarin	6.378 ± 0.897	10.280 ± 2.475	3.309 ± 0.325	2.207 ± 0.164
Daidzein	4.403 ± 1.002	15.877 ± 9.303	8.128 ± 2.664	14.425 ± 5.141
Genistein	0.517 ± 0.138	1.410 ± 1.003	0.327 ± 0.098	0.342 ± 0.035
Biochanin A	0.026 ± 0.007	0.085 ± 0.057	0.030 ± 0.014	0.028 ± 0.005

The literature showed that the bioavailability of glycoside compounds is low, but isoflavone glycosides such as puerarin (daidzein-8-C-glucoside) and daidzin (daidzein 7-O-glucoside) can be biotransformed into aglycones including daidzein by gut microbiota (Nakamura et al., 2020). Since aglycones were produced from *in vivo* metabolization and considering the decrease of puerarin and its glycosides while daidzein’s amount increased, the results indicated that puerarin and its glycosides probably acted as pro-drugs for M3 receptor inhibition. Although biochanin A and genistein were potent inhibitors for carbachol-induced detrusor contraction among these isoflavones, their amounts in Gegen water extract and urine were too low (less than 0.1%). Thus, for the *in vivo* inhibition against the M3 receptor, daidzein was the major active form for OAB, while puerarin, its glycosides, and daidzin were the pro-drugs.

Isoflavones such as daidzein have been reported in several studies through different mechanisms such as hormone replacement (Waetjen et al., 2013), anti-oxidant, and anti-inflammatory (Wu et al., 2018). Thus, our study provided more information on isoflavone-rich herbs in treating OAB with different etiology characteristics.

### 3.4 Thermal shift assay

**FIGURE 5:**
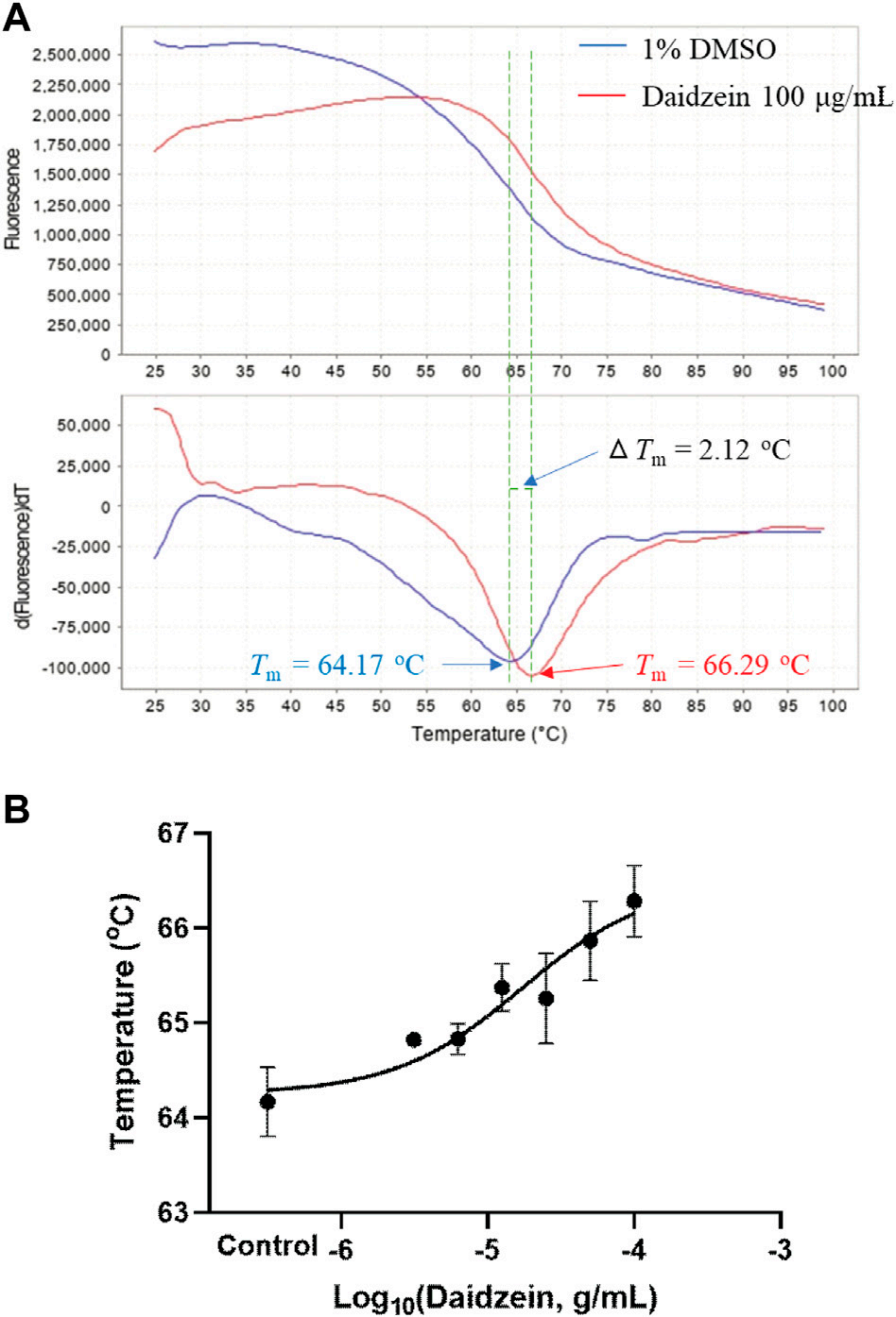
Ligand screening with the recombinant human M3 receptor protein as detected by thermal shift assay. **(A)** Representative fluorescence melt curve (upper) and derivative melt curve (lower) containing mean melt temperatures (*T*m) of the M3 receptor protein with or without daidzein (100 μg/ml), showing that binding of daidzein increased the thermal stability of the M3 receptor protein with Δ*T*m > 2 °C. **(B)** Daidzein (3.125–100 μg/ml) concentration-dependently increased the melt temperatures of the M3 receptor protein. Data are presented as Mean ± SEM (n = 3). DMSO (1%) served as the vehicle control.

As suggested by the supplier, the recombinant human M3 receptor protein can bind potent antagonists and be used for inhibitor screening. This membrane fraction contains BSA and glycerin; thus, daidzein was first checked for its possible binding to BSA. As a result, Δ*T*m of daidzein at 100 μg/ml was about 0.58°C in PBS solution containing BSA and glycerin at the same concentrations as the recombinant human M3 receptor protein. Furthermore, as shown in Figure 5A, daidzein at 100 μg/ml shifted the melt curve and increased the *T*m value of the human M3 receptor protein from 64.17 ± 0.37°C to 66.29 ± 0.38°C (*p* < 0.01). Since Δ*T*m was 2.12°C, daidzein was positively bound to the human M3 receptor protein as suggested (Huynh and Partch, 2015). In Figure 5B, daidzein concentration-dependently increased the *T*m values with an EC_50_ of 15.8 μg/ml at 65.3°C. *In vitro* thermal shift assay with the M3 receptor protein showed that daidzein was much more potent than the *ex vivo* detrusor contraction. At the same time, the *T*m value of the human M3 receptor protein interacted with puerarin at 100 μg/ml was 64.92 ± 0.03°C, showing its very weak binding to the M3 receptor protein.

### 3.5 Molecular docking analysis

Molecular docking results show that biochanin A, daidzein, genistein, 3′-hydroxypuerarin, 3′-methoxypuerarin, puerarin, daidzin, genistin, and puerarin apioside bound to the M3 receptor crystal structure (PDB ID 5ZHP) with different affinities (Table 3). However, only biochanin A, daidzein, and genistein showed potent binding free energy to the M3 receptor with a binding free energy lower than −7 kcal/mol (a basic binding free energy for the positive ligand–receptor interaction), which was consistent with the *ex vivo* results.

**TABLE 3:** Binding affinities (logarithm) of major components in Gegen water extract to the binding pocket of M3 receptor (PDB ID 5ZHP chain B).

Ligand	Binding free energy (kcal/mol)
9EC^a^	−11.5
Biochanin A	−9.6
Daidzein	−9.3
Genistein	−9.1
3′-Hydroxypuerarin	−5.7
3′-Methoxypuerarin	−5.0
Puerarin	−4.7
Daidzin	−2.8
Genistin	−2.8
Puerarin apioside	1.5

a M3 receptor (PDB ID 5ZHP) co-crystallized ligand 9EC: (1R,2R,4S,5S,7s)-7-({[4-fluoro-2-(thiophen-2-yl)phenyl]carbamoyl}oxy)-9,9-dimethyl-3-oxa-9-azatricyclo[3.3.1.0∼2,4∼]nonan-9-ium.

**FIGURE 6:**
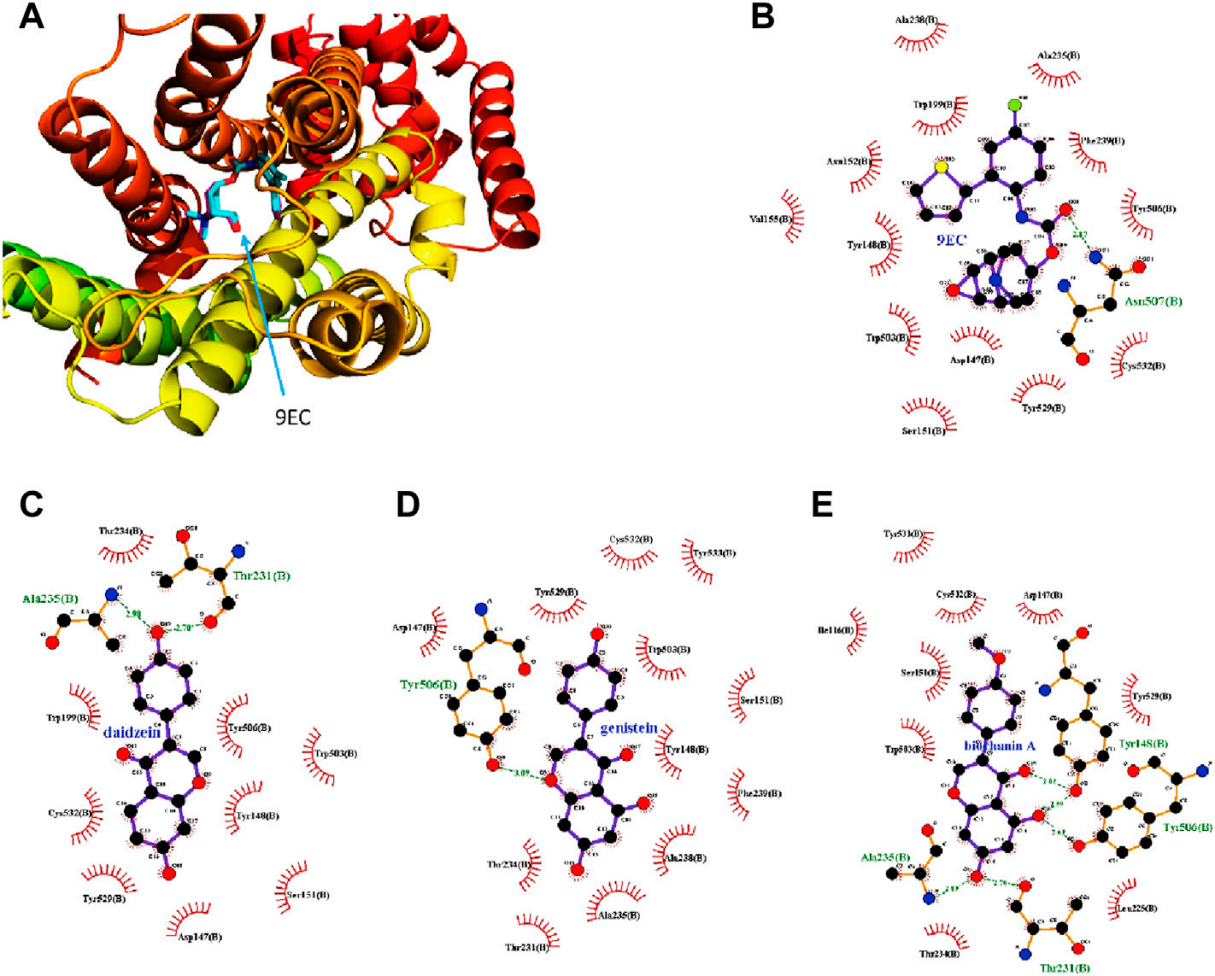
Molecular docking analysis indicates the best binding positions of the ligands with the lowest binding free energy in the ligand binding pocket of the M3 receptor (PDB ID 5ZHP chain B). The three-dimensional diagram illustrates the interactions of redocked 9EC (purple sticks) and the crystallized one (blue sticks) with the M3 receptor with the same binding position **(A)** which shows the high accuracy of the current docking method. The two-dimensional diagrams show the interactions of 9EC **(B)**, daidzein **(C)**, genistein **(D)**, and biochanin A **(E)** with the amino acid residues in the binding pocket of the M3 receptor. The spoked arcs show amino acid residues providing nonbonded interactions with the ligand. Green arrows with respective distances represent H-bonding between ligands and specific amino acid residues.

For docking simulation, the 3D conformation of the newly docked 9EC was highly consistent with the 3D conformation of the original co-crystal compound, indicating the high accuracy of this molecular docking (Figure 6A). Two-dimensional conformation analysis revealed that crystallized ligand 9EC strongly interacted with Asn507 through H-bonding at a distance of 2.87 Å and hydrophobic interactions with TRP199, PHE239, TYR506, ASP147, and TYR148, etc., and re-docked 9EC showed similar interactions (Figure 6B). Biochanin A, daidzein, and genistein have strong interactions with amino acid residues in the M3 receptor. For daidzein, its hydroxyl group in Ring C interacted with Thr231 and Ala235 through H-bonding (Figure 6C). For genistein, the ether group in Ring B interacted with TYR506 through H-bonding (Figure 6D). For biochanin A, hydroxyl groups at C-5 and C-7 in Ring A interacted with ALA235, THR231, and TYR506 through H-bonding, respectively, as did the ketone group in Ring B, which interacted with TYR148 (Figure 6E).

### 3.6 Structure–activity relationship

According to the inhibitory activities (IC values) of tested compounds on *ex vivo* carbachol-induced detrusor contraction as shown in Table 3, structure–activity relationships of isoflavones and their glycosides tested in the current study were analyzed and summarized as follows: first of all, puerarin (daidzein-8-C-glucoside) and its derivatives have lower inhibitory activities among these compounds. Substitution with a methoxyl group or hydroxyl group at C-3′ in Ring C could reduce M3 receptor inhibition, as well as substitution by glucose or apioside in Ring A. This is probably due to the reduced binding affinity to the active pocket of the M3 receptor. For instance, 3′-methoxypuerarin (inhibition rate at 100 μg/ml: 19.89 ± 3.38%) containing a methoxyl group and 3′-hydroxypuerarin (inhibition rate at 100 μg/ml: 17.75 ± 1.75%) at C-3′ showed weaker inhibitory activity than puerarin (inhibition rate at 100 μg/ml: 33.83 ± 3.05%). Genistin (Genistein-7-O-glucoside) (IC_50_ > 100 μg/ml) containing glucose and puerarin apioside containing apioside (inhibition rate at 100 μg/ml: 10.7 ± 9.24%) in Ring A showed weak inhibition on the M3 receptor. Second, when compared to daidzein (IC_50_: 40.27 ± 0.46 μg/ml), puerarin (inhibition rate at 100 μg/ml: 33.83 ± 3.05%) with substitution by glucuronic acid at C-5 in Ring A showed a weaker inhibitory effect on the M3 receptor; without substitution at C-8 in Ring A, daidzein (IC_50_: 40.27 ± 0.46 μg/ml) showed a stronger inhibitory activity. Compounds substituted with a hydroxyl group at Ring A showed stronger M3 receptor inhibition, which is probably due to the interaction with amino acid residues in the binding pocket of the M3 receptor. For example, the substitution of a hydroxyl group at the C-5 site in Ring A increased the inhibitory effect. Genistein (5-hydroxyl daidzein) (IC_50_: 18.57 ± 11.20 μg/ml) showed the strongest inhibitory effects among these compounds, whose substitution with the hydroxyl group at the C-5 site provided stronger binding to the M3 receptor when the ether group in Ring B interacted with TYR506 through a hydrogen bond. However, the substitution of a methoxyl group instead of a hydroxyl group at the C-4′ site in Ring C reduced the inhibitory activity. Biochanin A (genistein 4′-methyl ether) (IC_50_: 57.07 ± 12.47 μg/ml) with a methoxyl group showed a weaker inhibitory activity than genistein.

## 4 Conclusion

As pro-drugs, puerarin, its derivatives, and daidzin were the major chemical components of Gegen water extract, and daidzein was their major *in vivo* active form for M3 receptor inhibition against OAB at the threshold concentration in urine. It could probably act on the bladder wall since it reached an effective concentration in urine. Biochanin A and genistein were much more potent, but their contents were too low for the pharmacological effect of Gegen water extract. Due to the threshold effects of daidzein, other molecular mechanisms should be revealed to fully understand the underlying mechanisms of Gegen water extract against OAB in the future.

## Data Availability

The original contributions presented in the study are included in the article/Supplementary Material; further inquiries can be directed to the corresponding author.

## Abbreviations

OAB, overactive bladder; HPLC, high-performance liquid chromatography; SHR, spontaneously hypertensive rats; UV, ultraviolet; MS, mass spectrum; TQS, triple quadrupoles; *T*m, melt temperature; MRM, multiple reaction monitoring.
