# Supplementary material for: Daidzein is the in vivo active compound of Puerariae Lobatae Radix water extract for muscarinic receptor-3 inhibition against overactive bladder
Source: Front Pharmacol. 2022 Oct 4;13:924251. doi: 10.3389/fphar.2022.924251 (PMC9576955; doi:10.3389/fphar.2022.924251)
Supplement: Supplementary file 1 [file DataSheet1.docx]

Supplemental data

Daidzein is the *in vivo* active compound of Gegen (Puerariae lobatae Radix) water extract for muscarinic receptor-3 inhibition against overactive bladder

Yining Qiang^1^, Lu Bai^1^, Shuran Tian^1^, Yi Ma^1^, Pingxiang Xu^1,2^, Mingchang Cheng^1^, Yi Wu^1^, Xiaorong Li^1,2^, Ming Xue^1,2^, Xuelin Zhou^1^^,2,*^

^1^ Department of Pharmacology, School of Basic Medical Sciences, Capital Medical University, Beijing, 100069, China

^2^ Beijing Engineering Research Center for Nerve System Drugs, Beijing, 100053, China

*: Corresponding author:

Dr. Xuelin Zhou, peterxlzhou@gmail.com; zhouxuelin@ccmu.edu.cn

Figure S1 HPLC-UV chromatograms of (A) Gegen water extract (B) Authentic standards (C) F1-5, (D) F1-6, (E) F2-3, (F) F2-4 and (G) tube 8 from F2-5 which showed obvious inhibition effects in the *ex vivo* carbachol-induced detrusor contraction.

(A) Gegen water extract





(B) Authentic standards





(C) F1-5





(D) F1-6





(E) F2-3





(F) F2-4





(G) tube 8 from F2-5





Figure S2 Representative ions chromatograms of (A) puerarin (B) daidzein (C) genistein and (D) biochanin A at each lower limit of quantitation (LLOQ).


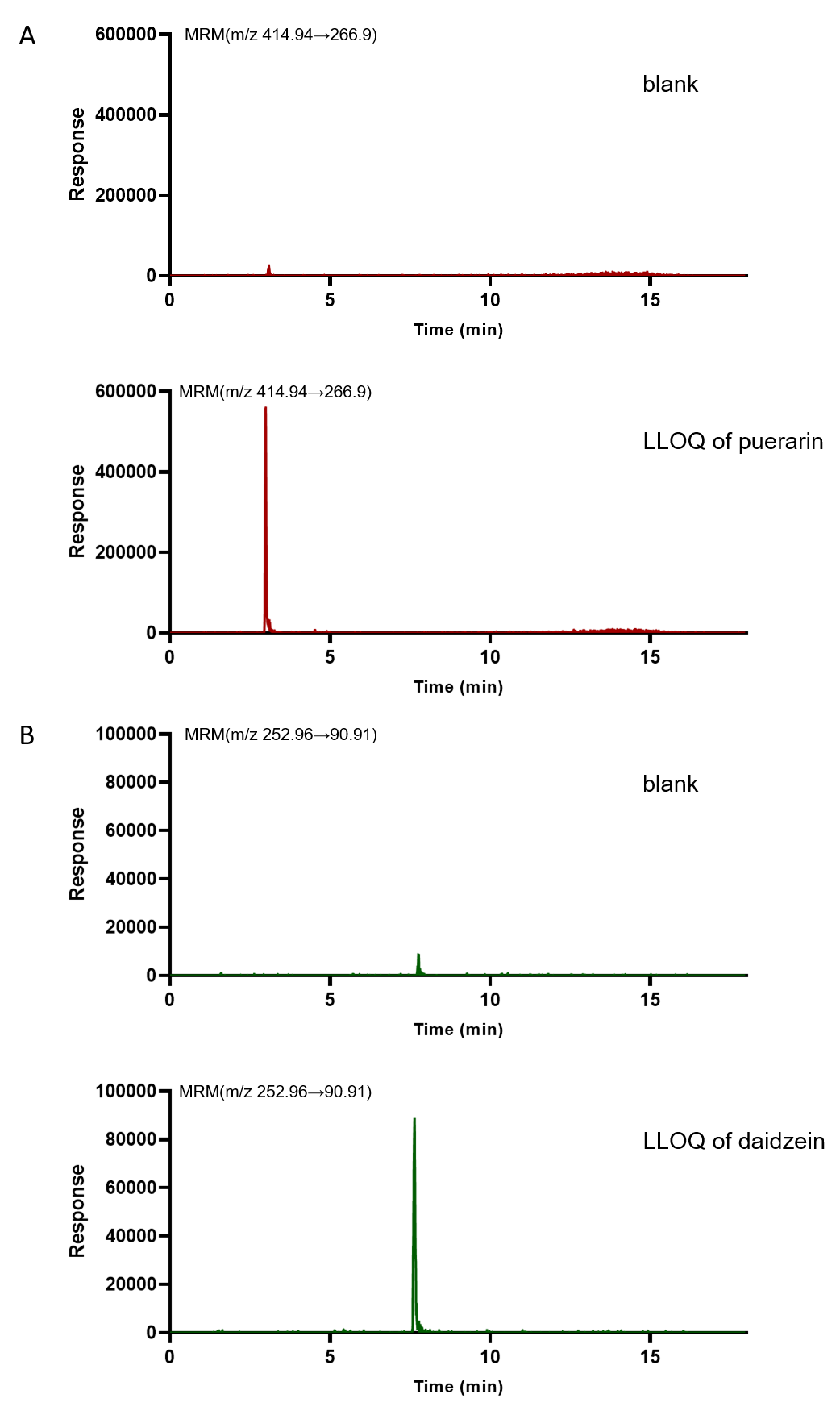


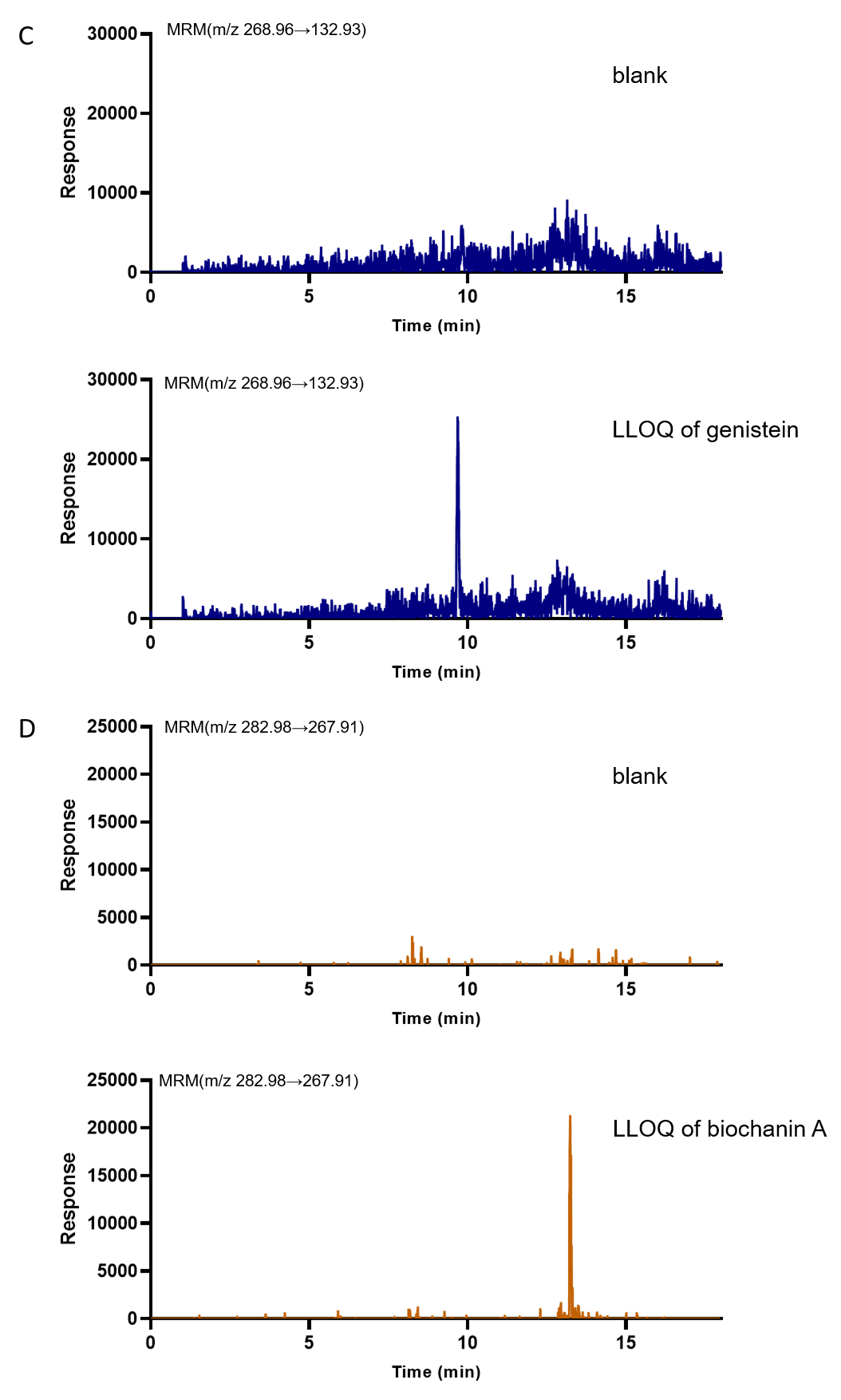


Figure S3 Immunochemical staining of M3 receptor in the bladder of (A) SHR, (B) Gegen (75mg/kg, p.o., 3-week) and (C) Gegen (300mg/kg, p.o., 3-week). Scale bars: 20 μm (n=6)


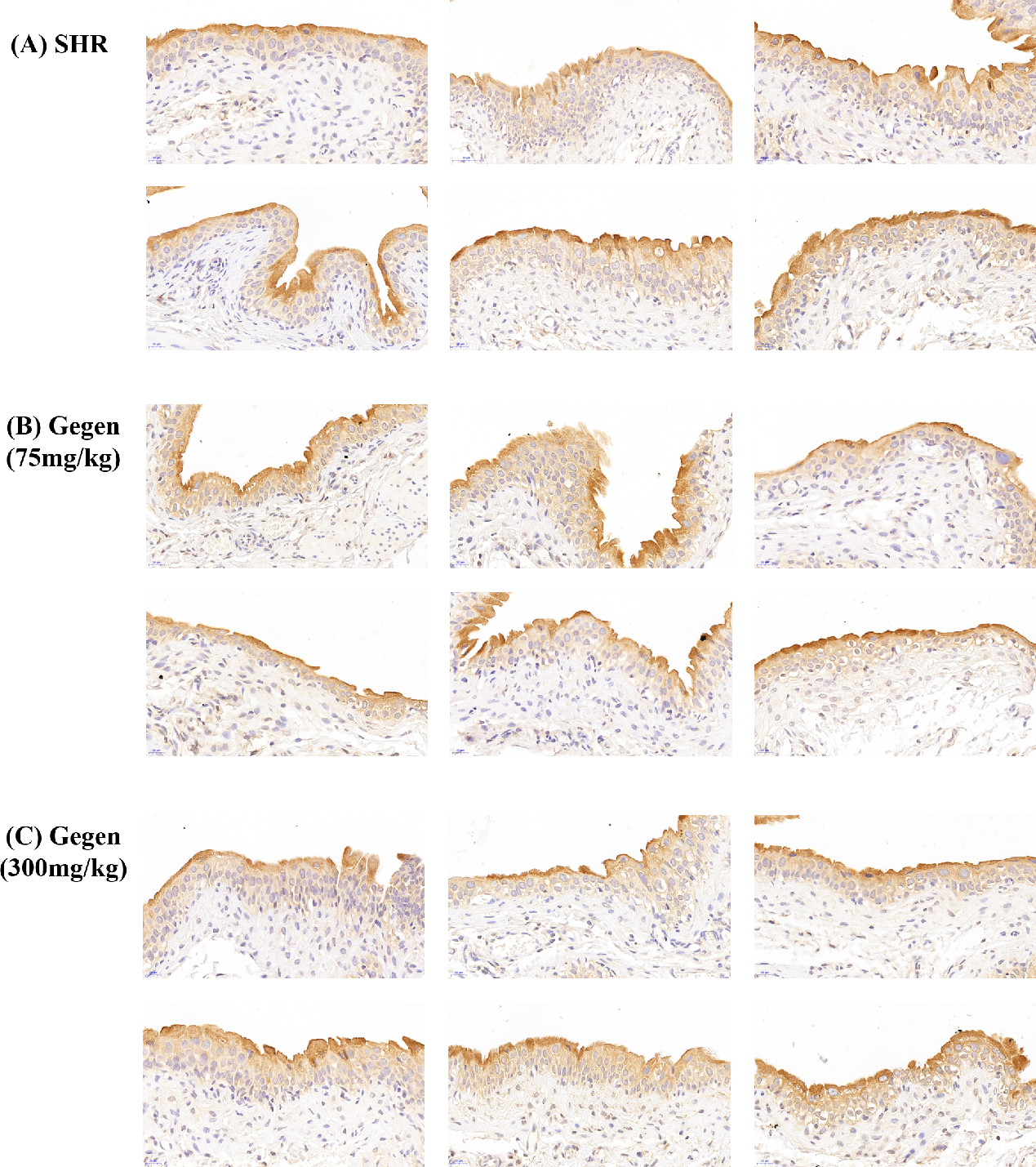


20 μm
